# The Longitudinal Relation between Observed Maternal Parenting in the Preschool Period and the Occurrence of Child ADHD Symptoms in Middle Childhood

**DOI:** 10.1007/s10802-018-0492-9

**Published:** 2018-11-12

**Authors:** Vandhana Choenni, Mijke P. Lambregtse-van den Berg, Frank C. Verhulst, Henning Tiemeier, Rianne Kok

**Affiliations:** 1 Department of Child and Adolescent Psychiatry/Psychology, Erasmus University Medical Center, Rotterdam, The Netherlands; 2 Department of Psychiatry, Erasmus University Medical Center, Rotterdam, The Netherlands; 3 Child and Adolescent Mental Health Center, Mental Health Services, Capital Region of Denmark, Copenhagen, Denmark & Faculty of Health and Medical Sciences, Department of Clinical Medicine, University of Copenhagen, Copenhagen, Denmark; 4 Department of Social and Behavioral Science, Harvard TH Chan School of Public Health, Boston, MA USA; 5 Department of Psychology, Education, & Child Studies, Erasmus University Rotterdam, Burg. Oudlaan 50, 3062 PA Rotterdam, The Netherlands

**Keywords:** Attention-deficit hyperactivity disorder (ADHD), Parenting, Comorbidity, Oppositional-defiant disorder (ODD), Etiology

## Abstract

In this longitudinal population-based cohort (*N* = 547) we examined the relation between maternal discipline and sensitivity in the preschool period and the occurrence of attention-deficit hyperactivity disorder (ADHD) symptoms in middle childhood, taking into account pre-existing child attention and executive function (EF) problems, and oppositional defiant disorder (ODD) symptom comorbidity. Maternal parenting was observed during a ‘do not touch task’ (positive and negative discipline) and a teaching task (sensitivity) at age 3. Parents reported on the occurrence of ADHD and ODD symptoms at age 8 using the Conners’ Parent Rating Scale. Attention and executive function problems were assessed using parent questionnaires at age 4. Important covariates such as harsh discipline and maternal depression were also taken into account. Maternal sensitivity significantly predicted later ADHD symptoms beyond pre-existing child attention and EF problems, and comorbid ODD symptoms. However, maternal negative and positive discipline did not significantly predict later ADHD symptoms over and above these covariates. This study demonstrates the importance of maternal sensitivity in the etiology of core ADHD symptoms above and beyond pre-existing child attention and EF problems, and comorbid ODD symptoms.

Attention-deficit hyperactivity disorder (ADHD) is the most common behavioral disorder in children and tends to persistently impact multiple areas of development into adulthood (e.g., Erskine et al. 2013; Kessler et al. 2005). Studies of the etiology of ADHD have often taken a biological viewpoint. For example, a genetic basis of ADHD has been demonstrated by twin studies on the heritability of ADHD (Faraone et al. 2005). Furthermore, the effectiveness of pharmacological treatment with methylphenidate is seen as further support for a neurobiological basis of ADHD (Storebø et al. 2015). However, there is growing evidence that environmental factors, specifically parenting, are involved in the etiology and, in particular, the persistence of ADHD (Deault 2010; Modesto-Lowe et al. 2008). In the current study, we investigate the predictive role of both positive and negative parenting during the preschool period on the occurrence of ADHD symptoms in middle childhood in a population-based cohort. For this purpose, we use elaborate observational measures of parenting practices during multiple structured mother-child interactions. We investigate whether several aspects of maternal parenting uniquely contribute to ADHD symptoms over and beyond pre-existing behavioral correlates of ADHD (i.e. attention and executive function problems) and comorbid symptoms of oppositional-defiant disorder (ODD).

In the developmental-transactional framework of ADHD etiology, parent-child interactions are placed at the center, illustrating how parenting reciprocally interacts with parent (e.g., depression) and child (e.g., disruptive behavior) characteristics and how it serves as a ‘filter’ through which other family variables (e.g., sibling relationships) influence child functioning, while taking into account the developmental stage of the child and the family (Johnston and Chronis-Tuscano 2015). This framework recognizes parenting as a central environmental factor which interacts with the child’s genetic predisposition, for example through epigenetic alterations, thus proposing parenting as an important dynamic etiological factor.

Across studies *acceptance-responsiveness* and *restrictiveness* are viewed as two core elements of parenting (Rothbaum and Weisz 1994). A key aspect of the acceptance-responsiveness dimension is sensitivity, the ability to perceive and to interpret accurately the child’s behavioral signals and to respond to these signals promptly (Ainsworth et al. 1978). Discipline, the capability to direct and manage child behavior, is a significant aspect of the restrictiveness dimension. Parental discipline can be further divided into positive discipline, characterized by parental support and empathy, and negative discipline, characterized by coercion and physical discipline techniques (van Zeijl et al. 2007). Extreme forms of negative discipline can overlap with harsh parenting, but the latter is a distinct aspect of parenting and should be distinguished from other parenting aspects.

The association between early parental restrictiveness and ADHD symptoms in (middle) childhood has been demonstrated in several longitudinal population-based studies. For example, observed maternal restrictiveness in infancy is related to more impulsive behavior in later childhood (Olson et al. 2002). Several other studies have also shown that high levels of self-reported early maternal restrictiveness (e.g., coercion, hostility, overreactivity, and inconsistent discipline) are related to attention, hyperactivity, and impulsivity problems/symptoms in later childhood (Breaux and Harvey 2018; Gadeyne et al. 2004; Galéra et al. 2011; Hawes et al. 2013; Romano et al. 2005). Children who exhibit ADHD symptoms likely deal with negative exchanges as a result of their inattentive and/or hyperactive behavior, which is difficult for parents to deal with (Pelham et al. 2004). According to coercive cycle theory (Patterson 2002), negative exchanges between parents and children can escalate, providing an explanation for the association between parenting and (the persistence of) ADHD symptoms.

Compared to the association between restrictiveness and ADHD symptoms, the association between acceptance-responsiveness and ADHD symptoms is less well studied. Higher levels of maternal sensitivity during structured play at preschool age predict better attentional control at age 6 (Belsky et al. 2007). Observed maternal sensitivity is also associated with father-rated attention problems (Keown 2012). Furthermore, observed maternal positive regard (e.g., positive affirmation and affection) is associated with mother−/father-reported attention problems and hyperactivity. Thus, higher levels of maternal sensitivity are prospectively related to less attention problems. An explanation for these results can be found in studies which show that normal variations in maternal sensitivity are associated with differences in child brain development (Kok et al. 2015; Sethna et al. 2017).

When studying the association between parenting and ADHD, it is important to take into account the considerable comorbidity between ADHD and symptoms of ODD (Bendiksen et al. 2014; Costello et al. 2003), such as angry and irritable mood or argumentative and defiant behavior. It is unclear whether parenting practices, specifically restrictiveness, are differentially related to ADHD symptoms as opposed to ADHD with comorbid ODD symptoms (Johnston and Mash 2001; Modesto-Lowe et al. 2008). More specifically, the observed association between restrictiveness and ADHD symptoms might be contributed to the comorbidity with ODD symptoms. For example, different aspects of restrictiveness, including negative/ineffective discipline, are related to the development and persistence of comorbid ODD symptoms in children with ADHD symptoms (August et al. 1999; Pfiffner et al. 2005). These studies suggest that restrictiveness is more strongly related to comorbid ODD symptoms than to ADHD symptoms. However, in one study maternal self-reported inconsistent discipline was associated with ADHD, after controlling for ODD and other symptoms (Ellis and Nigg 2009). This suggests that restrictiveness is associated with ADHD regardless of ODD symptoms.

Studies have increasingly highlighted the transactional relationship between parenting and ADHD symptoms over time, exploring parent-to-child as well as child-to-parent effects. In a sample of school-aged children, maternal control in kindergarten was associated with attention problems one year later and vice versa (Gadeyne et al. 2004). On the other hand, in two other studies child ADHD symptoms at age 11 predicted maternal rejection and hostility at age 12, while controlling for the stability of ADHD symptoms and maternal parenting over time (Lifford et al. 2008, 2009), but no evidence was found for a pathway from maternal parenting to child ADHD symptoms across time.

Often conclusions on the direction of effects are impeded by the possibility of reverse causation (i.e., child-to-parent effect). However, by controlling for initial levels of ADHD symptoms, the direction of effects becomes clearer and the contribution of parenting behaviors over and above existing child symptoms can be explored. For this reason, in the current study two well-known precursors of ADHD, EF and attention problems, are taken into account (Barkley 1997; Willcutt et al. 2005). These precursors were selected since ADHD symptoms cannot be assessed (by questionnaire) during the preschool period.

The aim of the current study is to examine the association between observed maternal sensitivity and discipline in the preschool period and ADHD symptoms in middle childhood in a non-clinical population. To study the influence of early parenting on later ADHD symptoms, early precursors of ADHD, specifically EF and attention problems, and comorbid ODD symptoms are taken into account. Additionally, we control for harsh discipline, to differentiate the association between normal variation in negative discipline and ADHD from effects of extreme or abusive discipline (Straus et al. 1998). Lastly, we control for maternal depression, because of its relation to both maternal parenting as well as ADHD symptoms in children (Cheung et al. 2018; Lovejoy et al. 2000). Many previous studies have used self-report on parenting practices or attitudes, which is only a proxy for actual parenting behavior (Holden and Buck 2002). In this study, elaborate measures of observed maternal parenting are used as direct and independent measures of parenting behavior in order to avoid reporting bias.

Based on previous findings and theoretical models we hypothesize that more maternal sensitivity and positive discipline, as well as less maternal negative discipline, are related to less ADHD symptoms. We expect that the associations between maternal parenting and child ADHD symptoms are independent of pre-existing child attention and EF problems, comorbid ODD symptoms, and covariates such as harsh discipline and maternal depression.

## Method

### Setting

The study was embedded in the Generation R Study, a prospective population-based cohort study investigating growth, development, and health from fetal life onwards in Rotterdam, the second largest city in the Netherlands (Jaddoe et al. 2010). The study area is defined by postal codes and covers more than half of the city’s inhabitants. Mothers with a delivery date between April 2002 and January 2006 were eligible and were informed about the study by midwives and obstetricians at their first prenatal visit. Thereafter, the study staff contacted these mothers to provide additional information and to obtain informed consent. Written informed consent was obtained from all adult participants. Detailed measurements were obtained in a subgroup of children of Dutch national origin (the Generation R Focus Study cohort), meaning that the children, their parents, and their grandparents were all born in the Netherlands (e.g., Luijk et al. 2010). The study was approved by the Medical Ethics Committee of the Erasmus Medical Center, Rotterdam.

### Study Population

For a total of 584 children, data specifically on observed maternal parenting at the 3-year visit and parent report on ADHD symptoms at age 8 was available. Thirty-seven families were excluded due to technical or procedural difficulties during observations or due to participation with twins. The final sample for analysis consisted of 547 unique mother-child dyads. The mother-child dyads excluded from the analyses did not differ on any of the background variables, covariates, or main outcomes. Descriptive statistics are provided in Table 1. Child sex, maternal age, and educational level were reported at study intake. Educational level was dichotomized as “high” (68%; at least higher vocational training or a bachelor’s degree) and “low/medium” (32%).

**Table 1:** Sample characteristics (*N* = 547)

Mother	
Age at intake (years)†	31.9 (3.7)
Educational level, %high^a^	67.6
Maternal depressive symptoms^b^	0 (0–0.17)
Positive discipline	0.14 (−0.89–1.05)
Negative discipline	−0.25 (−1.05–0.75)
Sensitivity†	0.01 (0.7)
Child	
Age at ADHD report (years)†	8.08 (0.13)
Sex, %female^a^	48.3
Attention problems 3 years^c^	1 (0–2)
Executive function problems 4 years^d^	45 (40–52)
ODD symptoms 8 years	3 (1–5)
ADHD symptoms 8 years	5 (2–11)

Unless otherwise indicated, median (*IQR*) of the untransformed variables in the original dataset are reported

^a^Percentage of total sample

^b^*N* = 523

^c^*N* = 519

^d^*N* = 515

† Values are mean (*SD*)

### Central Measures

#### ADHD and ODD Symptoms

The occurrence of ADHD and ODD symptoms was assessed with parent-report at age 8 using the Dutch version of the Conners’ Parent Rating Scale-Revised: Short Form (CPRS-R:S; Conners 1997). In this 27-item questionnaire, the primary caregiver (90% mothers) reports on child problem behavior in the past month on four scales; ADHD, Cognitive Problems/Inattention, Hyperactivity, and Oppositional behavior. All items are rated on a 4-point scale, ranging from 0 (*not true at all*) to 3 (*very much true*). Higher scores indicate more ADHD-related behaviors. The scores on the ADHD subscale (12 items), for example ‘short attention span’ and Oppositional subscale (6 items), for example ‘loses temper’, were used for this study. Composite scores for ADHD and ODD were positively skewed and square root transformed to approach normality. In the current study sample, high internal consistencies were found for the total scale, ADHD subscale, and Oppositional subscale (respectively, Cronbach’s *α =* 0.94; *α =* 0.91; *α =* 0.81).

#### Maternal Parenting

Maternal discipline was observed at a laboratory visit around age 3 (*M* = 3.13, *SD* = 0.13) during a 2-min task (“Do not touch”) in which the mother prohibited the child to touch or play with a set of attractive toys. Maternal verbal and physical discipline strategies were coded in different categories: Commands, Support, and Physical Obstruction/interference (coded per statement or instance; Kuczynski et al. 1987; van der Mark et al. 2002); and with the revised Erickson 7-point rating scale for Supportive Presence (macro coded; Egeland et al., 1990). The coding procedure has been described in detail elsewhere (Kok et al. 2013). Reliability of the coders was assessed directly after training and at the end of the coding process to detect possible rater drift. The overall intraclass correlation coefficients (ICC) were as follows, for Commands 0.85, for Support 0.85, for Physical Interference 0.90, and for Supportive Presence 0.79 (*n* = 57). An overall maternal positive discipline score (i.e. Support and Supportive Presence) and an overall maternal negative discipline score (i.e. Commands and Physical Obstruction/interference) were created by standardizing and summing the scores. The positive discipline score was negatively skewed and the negative discipline score was positively skewed, both were transformed (resp. square root transformation and log transformation) to approach normality.

Maternal sensitivity was observed at age 3 during two 3- to 4-min teaching tasks (“Building a tower” and “Etch-a-Sketch”) that were too difficult to complete for the child independently. The “Building a tower” task contains a rod and a set of blocks that can be placed on the rod, but only in a particular order, as the blocks are like puzzle pieces that fit together in a certain way. The “Etch-a-sketch” is a drawing board with two knobs: if the child turns the left knob, a vertical line appears; if the child turns the right knob, a horizontal line appears. A maze was taped on the drawing board and the child needed to go from the start of the maze to the center of the maze, by alternately turning the two knobs. Mothers were instructed to help their child as usual when introducing a new toy. Maternal sensitivity was coded with the revised Erickson 7-point rating scales for Supportive Presence and Intrusiveness (Egeland et al., 1990). Reliability of the coders was assessed directly after training and at the end of the coding process to detect possible rater drift. The coding procedure has been described in detail elsewhere (Kok et al. 2012). The average ICC for the tower task was 0.75 (*n* = 53) and for the etch-a-sketch task 0.79 (*n* = 55). An overall sensitivity score was created by reversing the Intrusiveness scales, standardizing the scores on the subscales (Z-score), and creating an average over both subscales and both tasks. Correlations between supportive presence and intrusiveness within and across teaching tasks ranged from *r* = 0.16 to 0.39.

### Covariates

#### Maternal Depressive Symptoms and Maternal Harsh Parenting

The Dutch version of the Brief Symptom Inventory (Derogatis 1983) was used to assess maternal depressive symptoms at age 3. Scores were positively skewed and log-transformed to approach normality. Maternal depression was included as a covariate in all models. In the current sample, internal consistency of the depression subscale was high (Cronbach’s *α* = 0.75).

Mothers reported on their use of harsh discipline at the 3-year follow-up with the Parent-Child Conflict Tactics Scales (PC-CTS; Straus et al. 1998). Harsh parenting was positively skewed and square root transformed to approach normality. Since harsh parenting needs to be differentiated especially from negative discipline, it was added as a covariate specifically to these two regression models, but not to the sensitivity and positive discipline models. Internal consistency was poor in the current sample (Cronbach’s *α* = 0.51).

#### Child Attention and EF Problems

To explore the effect of maternal parenting beyond pre-existing child attention and EF problems on later child ADHD, these potential precursors were added in a separate step in the regression model. Attention problems were assessed via parent-report on the five items of the attention syndrome scale of the Dutch Child Behavior Checklist 1.5–5 at age 3 (CBCL; Achenbach and Rescorla 2002). This score was positively skewed and square root transformed to approach normality. Internal consistency for the attention problems subscale was moderate in the current study sample (Cronbach’s *α =* 0.64).

Executive function problems were assessed with the Dutch version of the Behavior Rating Inventory of Executive Function-Preschool Version at age 4 (BRIEF-P; Gioia et al. 2003). In this 63-item questionnaire, parents report on child EF domains, e.g., inhibition, shifting, and emotional control in the past month. A higher Global Executive Composite (GEC) T-score (adjusted for age group and sex) on the BRIEF-P represents more overall EF problems. The GEC was positively skewed and transformed inversely to approach a normal distribution, after which it was mirrored to facilitate interpretation. In the current study sample, internal consistency of the total scale was excellent (Cronbach’s *α =* 0.94).

### Statistical Analyses

Due to missing data on maternal educational level (*n* = 4), maternal depressive symptoms (*n* = 24) maternal harsh discipline (*n* = 27), child attention problems (*n* = 28), and child EF problems (*n* = 32) we generated 20 imputed datasets by multiple imputation. Based on between-group comparisons for the five aforementioned variables with missing data and Little’s MCAR Test, we found that these date were missing (completely) at random. Analyses conducted with the imputed datasets (*N* = 547) yielded similar results compared to the analyses with the complete dataset (*N* = 515). Results with the imputed dataset are presented unless otherwise indicated.

First, bivariate associations among maternal parenting, child ADHD symptoms, covariates, and background variables were explored with Pearson’s correlations and *t*-tests. Second, we performed three separate linear regression analyses to examine whether each of the three aspects of observed maternal parenting at age 3 (positive discipline, negative discipline, sensitivity) predicted ADHD symptoms at age 8, independent of pre-existing child attention and EF problems, comorbid ODD symptoms, maternal depressive symptoms, and background variables. Each model was composed of four blocks. In the first block, all covariates were entered. In the second block, child attention and EF problems were added. In the third block, comorbid ODD symptoms were added. In the final block, maternal parenting (i.e., sensitivity, positive discipline, or negative discipline) was entered. Third, a regression analysis including all three parenting variables in the final block (and covariates in the previous blocks) was performed, to isolate the impact of the individual elements of parenting. Lastly, sex differences in the association between observed parenting and ADHD were explored, by examining interactions between parenting and child sex on ADHD symptomatology.

## Results

A sample description is presented in Table 1. Boys showed more ADHD symptoms than girls, *t*(545) = 4.45, *p* < 0.001. Furthermore, mothers used more positive discipline strategies toward girls than toward boys, *t*(545) = −3.26, *p* = 0.001, and fewer negative discipline strategies toward girls than toward boys, *t*(545) = 4.24, *p* < 0.001. Similarly, mothers were more sensitive toward girls than toward boys, *t*(545) = −2.08, *p* = 0.038. Maternal age was not associated with maternal parenting or child ADHD symptoms. Highly educated mothers used more positive discipline than mothers with a lower educational level, *t*(541) = −2.19, *p* = 0.029, and highly educated mothers were more sensitive than mothers with a lower educational level, *t*(541) = −4.16, *p* < 0.001.

The bivariate correlations among the main variables and covariates are presented in Table 2.

**Table 2:** Correlations between child ADHD symptoms, maternal parenting, and covariates (*N* = 547)

	Negative Discipline	Sensitivity	Maternal depressive sym.	Attention pr. 3 yrs.	EF pr. 4 yrs.	ODD sym. 8 yrs.	ADHD sym. 8 yrs.
Positive discipline	−0.68**	0.20**	−0.06	−0.12**	−0.11**	−0.10*	−0.14**
Negative discipline		−0.26**	0.05	0.15**	0.11*	0.07	0.16**
Sensitivity			−0.06	−0.15**	−0.15**	0.01	−0.16**
Maternal depressive sym.				0.23**	0.23**	0.23**	0.18**
Attention pr.					0.47**	0.26**	0.34**
EF pr.						0.38**	0.41**
ODD sym.							0.43**

*Yrs.* years; *Sym*. symptoms; *EF* Executive Function; *Pr* problems

* *p* < 0.05, ** *p* < 0.01

Maternal sensitivity correlated with more positive discipline and less negative discipline at child age 3 years. There was a negative correlation between maternal positive and negative discipline. Furthermore, all maternal parenting measures were significantly correlated with attention and EF problems, and ADHD symptoms in the expected direction.

### Maternal Parenting and ADHD Symptoms

Table 3 shows that maternal sensitivity at age 3 significantly predicted less child ADHD symptoms at age 8, over and above covariates, early child attention and EF problems, and comorbid ODD symptoms. Maternal positive discipline did not predict child ADHD symptoms at age 8, after adjusting for covariates, pre-existing attention and EF problems, and ODD comorbidity (Table 4). The association between maternal negative discipline and child ADHD symptoms was at trend level (β = 0.07, *p* = 0.06) but not significant, after adjustment for the same covariates, pre-existing and comorbid symptoms (Table 5). The interactions between observed maternal parenting and sex on child ADHD symptoms were not significant (data not shown).

**Table 3:** Maternal sensitivity as a predictor of child ADHD symptoms at age 8 adjusted for early child EF and attention problems, and comorbid ODD symptoms (N = 547)

	*B* ^a^	*t*	*p*	*R* ^*2*a^
Block 1				0.08
Child sex	−0.12	−3.22	0.001	
Maternal age	0.03	0.74	0.459	
Maternal educational level	−0.06	−1.63	0.103	
Maternal depressive sym.	0.02	0.63	0.528	
Block 2				0.22
Attention pr.	0.13	3.09	0.002	
EF pr.	0.18	4.06	<0.001	
Block 3				0.30
Child ODD sym.	0.32	8.02	<0.001	
Block 4				0.30
Sensitivity	−0.09	−2.38	0.017	

*Sym*. symptoms; *EF* Executive Function; *Pr* problems

^a^Averages taken from the final regression models of the 20 imputed datasets

**Table 4:** Maternal positive discipline as a predictor of child ADHD symptoms at age 8 adjusted for early child EF and attention problems, and comorbid ODD symptoms (N = 547)

	*B* ^a^	*t*	*p*	*R* ^*2*a^
Block 1				0.08
Child sex	−0.12	−3.18	0.001	
Maternal age	0.04	0.83	0.407	
Maternal educational level	−0.07	−1.93	0.053	
Maternal depressive sym.	0.02	0.63	0.526	
Block 2				0.22
Attention pr.	0.14	3.19	0.001	
EF pr.	0.19	4.26	<0.001	
Block 3				0.30
Child ODD sym.	0.30	7.74	<0.001	
Block 4				0.30
Pos. discipline	−0.04	−1.21	0.225	

^a^Averages taken from the final regression models of the 20 imputed datasets

*Sym*. symptoms; *Pos*. Positive; *EF* Executive Function; *Pr* problems

**Table 5:** Maternal negative discipline as a predictor of child ADHD symptoms at age 8 adjusted for early child EF and attention problems, and comorbid ODD symptoms (N = 547)

	*B* ^a^	*t*	*p*	*R* ^*2*a^
Block 1				0.08
Child sex	−0.11	−2.17	0.003	
Maternal age	0.04	0.79	0.430	
Maternal educational level	−0.07	−1.85	0.065	
Maternal depressive sym.	0.04	0.66	0.512	
Block 2				0.22
Attention pr.	0.13	3.06	0.002	
EF pr.	0.18	4.30	<0.001	
Block 3				0.30
Child ODD sym.	0.28	7.77	<0.001	
Block 4				0.30
Neg. discipline	0.07	1.85	0.064	

Data are not shown for the model including harsh discipline

^a^Averages taken from the final regression models of the 20 imputed datasets

*Sym*. symptoms; *Neg*. Negative; *EF* Executive Function; *Pr* problems

In the regression model including all three parenting predictors at once, maternal sensitivity again appeared as the main parenting predictor for child ADHD symptoms at age 8 (Table 6).

**Table 6:** Maternal sensitivity and discipline as predictors of child ADHD symptoms at age 8 adjusted for early child EF and attention problems, and comorbid ODD symptoms (N = 547)

Predictor	*B* ^a^	*t*	*p*	*R* ^*2*a^
Block 1				0.08
Child sex	−0.11	−2.97	0.003	
Maternal age	0.03	0.73	0.468	
Maternal educational level	−0.06	−1.55	0.122	
Maternal depressive sym.	0.02	0.64	0.525	
Block 2				0.22
Attention pr.	0.13	2.96	0.003	
EF pr.	0.19	4.1	<0.001	
Block 3				0.30
ODD sym.	0.32	7.92	<0.001	
Block 4				
Sensitivity	−0.08	−2.01	0.045	0.31
Neg. discipline	0.05	1.07	0.283	
Pos. discipline	0.00	0.08	0.939	

*Sym*. symptoms; *Pos*. Positive; *Neg*. Negative; *EF* Executive Function; *Pr* problems

^a^Averages taken from the final regression models of the 20 imputed datasets

All regression models were rerun with untransformed variables to check whether transformation or non-normality affected the results. All analyses yielded similar results, except for the regression model with negative discipline as predictor of ADHD symptoms, controlled for covariates. In the model with untransformed variables, negative discipline significantly predicted ADHD symptoms (β = 0.07, *p* = 0.04). In the model with transformed variables (see Table 5), the association between negative discipline and ADHD symptoms was at trend level (β = 0.07, *p* = 0.06).

## Discussion

In this population-based cohort study, we examined whether maternal sensitivity and positive and negative discipline at age 3 were related to child ADHD symptoms at age 8, over and above pre-existing child attention and EF problems, and comorbid ODD symptoms. We found that higher levels of maternal sensitivity at age 3 were significantly associated with less ADHD symptoms at age 8. While the association between maternal sensitivity and child ADHD symptoms was modest, it was independent of pre-existing child attention and executive function problems, comorbid ODD symptoms, and other covariates. This finding supports the hypothesis that maternal parenting, specifically sensitivity, contributes to the etiology of ADHD symptoms. Maternal negative and positive discipline did not predict ADHD symptoms after adjustment for the role of covariates, pre-existing child attention and EF problems, and comorbid ODD symptoms.

Our findings are in correspondence with previous studies on the association between child ADHD symptoms and maternal sensitivity and other aspects of acceptance-responsiveness. For example, Keown (2012) demonstrated an association between maternal sensitivity, positive regard, and ADHD, beyond existing hyperactivity symptoms and conduct problems. However, the study by Ellis and Nigg (2009) showed that maternal reports of involvement and positive parenting were not related to ADHD-symptoms. The discrepancy between these results can be best explained by methodological differences. In the latter study, maternal parenting was assessed via self-report, providing a less direct and independent measure of parenting. Moreover, maternal sensitivity is related to involvement and positive parenting, but encompasses other elements of acceptance-responsiveness as well, thus representing this broad spectrum more accurately. Along this line, it could be argued that maternal sensitivity and positive discipline are related as well, considering the shared aspects, such as positive regard toward the child. However, in our study positive discipline did not uniquely predict ADHD. Positive discipline (e.g., empathizing with frustration) was measured in a compliance task aimed at putting mild pressure on the parent-child interaction, while sensitivity was measured in a teaching task emphasizing the encouragement and support (e.g., giving praise and scaffolding) provided by the mother. It is plausible that specific situational demands (i.e., the pressure of needing to exert control to manage non-compliance) differentially elicit positive (or negative) discipline. Possibly, positive discipline is observed better in other situations, such as free play or a teaching task, with less pressure to enforce rules. Also, our operationalization of sensitivity was more comprehensive than the operationalization of positive discipline, increasing the possibility to find an association between maternal sensitivity and ADHD. Lastly, our findings are in line with studies on the association between parenting and precursors of ADHD. For example, more maternal sensitivity is associated with better executive functioning (Bernier et al. 2010; Kok et al., 2014) and better attentional control and regulation (Belsky et al. 2007).

The association between negative discipline and ADHD did not exceed trend level when adjusted for pre-existing EF and attention problems and comorbid ODD symptoms. This finding is in line with other studies in which negative discipline is more strongly associated with ADHD and comorbid ODD than with ADHD alone (e.g. August et al. 1999; Pfiffner et al. 2005). However, findings are mixed and some (non-clinical) studies have found an association between negative discipline and child ADHD symptoms (e.g. Ellis and Nigg 2009). It should be noted that all associations between parenting and ADHD symptoms in the current study were stringently corrected for multiple covariates, including pre-existing and comorbid symptoms, which could have limited the power to detect weak associations. The fact that the significance of the association between negative discipline and child ADHD symptoms was dependent on (transformation for) non-normality, indicates the need for future studies to shed more light on the relevance of normal variation in negative discipline for the etiology of ADHD symptomatology.

By stringently correcting for pre-existing attention and EF problems, we aimed to clarify the direction of the association between maternal parenting and ADHD and to consider the possibility of reverse causality. Although this correction for pre-existing ADHD-related problems cannot completely rule out reverse causality, we provided important evidence of the predictive additional value of maternal sensitivity for core ADHD symptoms.

Our study has several strengths. Firstly, we used detailed observational measures of maternal parenting in multiple mother-child interaction tasks. This is in contrast to most other longitudinal population studies, which commonly assess parenting with questionnaires. Also, we included less well-studied aspects of parenting, positive discipline and sensitivity, in addition to negative discipline. Furthermore, we stringently corrected for pre-existing attention and EF problems, and comorbid ODD symptoms which enabled us to demonstrate the effect of each parenting aspect above and beyond these problems. However, some limitations must be noted. Both parenting and ADHD symptoms were assessed at single time points. We are therefore not able to determine whether maternal parenting beyond the preschool period is related to ADHD symptoms in middle childhood. Moreover, multiple measurements of parenting (in different settings) and repeated follow-up measurements of ADHD (i.e., cross-lagged design), allow for greater insight into the transactional relationship between changes in parenting and changes in ADHD symptoms over time. Also, with regard to potential precursors of ADHD, we included attention and EF problems, but not symptoms of hyperactivity-impulsivity. In addition, we could not control for a shared genetic risk, effects of parental ADHD symptomatology or parental EF problems, as alternative factors explaining variation both in parenting behavior and in child ADHD symptoms (Deater-Deckard 2015; Park et al. 2017). Additionally, in the current study father-child dyads were excluded due to limited participation. However, maternal and paternal parenting have independent effects on children’s behavior in middle childhood (Scott et al. 2018). This warrants attention in future studies, which should extend our findings by studying the role of paternal parenting in child ADHD and by considering multi-informant ADHD symptom reports. Limited evidence to date illustrates that less paternal acceptance-responsiveness and more paternal restrictiveness are associated with higher levels of child ADHD symptoms, similar to the associations that are found for mothers (Chang et al. 2013; Keown 2012). Furthermore, other studies should shed light on possible mechanisms in the relation between parenting and child ADHD symptoms. For example, the extent to which children have the opportunity to practice and ‘adjust’ behavior and thereafter receive a sensitive parent response is an important factor for the development of adaptive or maladaptive behavior (Grolnick and Farkas 2002). Moreover, our results pertain to a general population sample. Future research within more diverse samples can test the generalizability of our results to high-risk or clinical populations.

## Conclusion

Our findings illustrate the unique contribution of maternal sensitivity, beyond pre-existing attention and EF problems, and ODD comorbidity, in the development of attention and hyperactivity problems in children. Although a modest association between maternal sensitivity and child ADHD symptoms was found, this finding further increases the understanding of the etiology of core ADHD symptoms. Also, it provides an avenue for the incorporation of parental sensitivity into psychoeducation or parental guidance programs aimed at ADHD symptomatology.
